# Association of serum osteoprotegerin with severity of chronic liver disease in female patients: A potential biomarker

**DOI:** 10.12669/pjms.36.6.2678

**Published:** 2020

**Authors:** Saba Tariq, Sundus Tariq, Shaista Hussain, Mukhtiar Baig

**Affiliations:** 1 Saba Tariq, MBBS, M.Phil. Associate Professor of Pharmacology, University Medical & Dental College, University of Faisalabad, Pakistan, Research Scholar, Pharmacology, University of Health Sciences, Lahore, Pakistan; 2 Sundus Tariq, MBBS, M.Phil. Associate Professor of Physiology, University Medical & Dental College, Faisalabad, Pakistan. Research Scholar, Physiology, University of Health Sciences, Lahore, Pakistan; 3 Shaista Hussain, MBBS. House Officer, Allied Hospital Faisalabad, Faisalabad, Pakistan; 4 Prof. Mukhtiar Baig, MBBS, M.Phil., PhD. Department of Clinical Biochemistry, Faculty of Medicine, Rabigh, King Abdulaziz University, Jeddah- 21589, KSA

**Keywords:** Osteoprotegerin, Chronic liver disease, liver cirrhosis, Hepatomegaly

## Abstract

**Objective:**

To determine the association of serum osteoprotegerin (OPG) with the severity of chronic liver disease in female patients.

**Methods:**

This case-control study was conducted in Madina Teaching Hospital from 2019-2020.An institutional review board of University Medical and Dental College, The University of Faisalabad gave the approval to conduct the study. Only female patients of age group 40 to 60 years having CLD were included in this study. Total 80 participants were enrolled after fulfilling the inclusion and exclusion criteria. Serum OPG levels were measured by enzyme linked immunosorbant assay (ELISA) supplied by ELAB Sciences, USA. The severity of disease was assessed by Child-Pugh classification.

**Results:**

OPG levels were significantly different between the three Child-Pugh classes. OPG levels were significantly high in class C indicating increased level of this cytokine in CLD as compared to class A (*p* = <0.05). There was a positive association of OPG with splenomegaly (OR = 2.10, *p* = <0.001), hepatomegaly (OR = 4.41, (*p* = <0.05), skin pigmentation (OR = 2.06, *p* = <0.05), malena (OR = 1.87, *p* = <0.05) and prolonged bleeding (OR = 1.86, *p* = <0.05).

**Conclusion:**

The levels of serum Osteoprotegerin is increased in severe form of chronic liver disease (Class C) of Child-Pughs classification as compared to mild (Class A) and moderate (Class B) forms of Child-Pughs classification.

## INTRODUCTION

The extent of public health burden of Chronic liver disease (CLD) is becoming so frequent that it is considered as one of the main cause of death in underdeveloped countries.^1^ The most frequently encountered causes of CLD includes infection with Hepatitis B and C virus, alcoholic liver disease and non-alcoholic fatty liver disease.^2^

The main mechanism for the progression of CLD is the inflammation of supporting network of liver resulting in hepatic scarring and disturbed architecture due to assembling of collagens and proteoglycans that leads to liver cirrhosis.^3^ Cirrhosis is one of the complication of CLD and can be characterized as the formation of small irregular non-neoplastic masses that are encircled by dense bands of fibrosis that leads to raised blood pressure in portal venous system and chronic liver failure.^4^

RANK, RANKL and OPG are the members of TNF superfamily and osteoprotegerin competes with RANKL for binding with RANK that result in its activation that are necessary to start several biochemical cascades.^5^ Osteoprotegerin to osteoclastogenesis inhibitory factor proportion is higher in those having CLD. Osteoprotegerin levels are elevated as a compensatory response to halt the bone loss which is common in CLD.^6^ A recent research shows, that there is up-regulation of gene expression of these members of TNF superfamily in serum of patients with jaundice that results in elevation of serum osteoprotegerin in patients of chronic liver disease.^7^

Data suggests that there is significant association between serum levels of osteoprotegerin and functions of albumin and prothrombin indicating that there is a correlation between increased levels of elevated osteoprotegerin and disturbed hepatic physiology.^8^ Sustained release of OPG in patients of CLD has been demonstrated that it must be due to the mediators involved in inflammation indicating that these members of TNF superfamily perform a modulatory action in inflammation.^9^ Another study shows that there is a correlation between serum osteoprotegerin levels in patients having hepatitis C virus infection of prolonged duration with many cardiovascular complications.^10^

As chronic liver disease is one of the most prevalent diseases in Pakistan therefore the present study was designed to see relationship of serum OPG with severity of chronic liver disease in female patients.

## METHODS

The present study was conducted in Madina Teaching Hospital, Faisalabad. This case-control study was conducted in the year 2019-2020. An institutional review board of University Medical and Dental College, The University of Faisalabad gave the approval to conduct this study according to Helsinki Declaration of Human Rights vide letter no (TUF/Dean/2019/47). Madina Teaching Hospital is a tertiary care hospital and have access to large number of patients. A written informed consent was taken from all subjects participating in the study. Only female patients of age group 40 to 60 years were included in the study. Sample size was calculated using openEpi calculator. The calculated sample size for cases was 47 and for controls was 14 using confidence level of 95%, power of study 80%, ratio of controls to cases 0.29, hypothetical proportion of controls with exposure 40, proportion of cases with exposure 79.42, and least extreme odds ratio to be detected 5.79.^11^ Total 80 participants were enrolled in this study; they were categorized into two groups. First group had 50 participants that were having the chronic liver disease due to hepatitis C confirmed through PCR and second group (control) included 30 participants. Patients were included having the evidence of chronic liver disease on evaluation by the consultant physician. Participants with morbid obesity, renal failure, alcoholic liver disease and osteoporosis were excluded from the study as they can interfere with the OPG levels.

From all participants, information was collected on a specially designed proforma. Information was collected related to age and signs and symptoms of CLD. Disease severity was assessed by Child-Pugh’s classification. Child Pugh’s classification is used to determine the severity of liver cirrhosis. There are three major classes of Child Pugh’s classification, to categorize the patients into three different classes several different clinical parameters are used and scoring is made on the basis of these parameters. Patients having a score of 5 or 6 are assigned to class A, patients with scores 7–9 are categorized to class B, and patients with scores 10–15 are categorized to class C.^12^,^13^

Anthroprometric data was obtained with the help of standard method and general physical examination was performed by trained physician. Blood samples were taken early in the morning (12-14 hour fasting) and were centrifuged at 3000 rpm for 10 minutes within the first hour of collection, and the isolated serum samples were stored at -80°C until assayed. OPG levels were measured by enzyme linked immunosorbant assay (ELISA) supplied by ELAB Sciences, USA.

The data was entered and analyzed using SPSS 20. Mean (SD) was given for quantitative continuous variable. Normality of the data was checked by shapiro-wilk statistic. Comparisons between cases and controls was seen using independent sample t-test. Analysis of variance (ANOVA) was used to see the association of disease severity (Child-Pugh classification) with serum OPG levels. Tukey’s post-hoc analysis was used to see individual differences between the groups. Comparison of OPG levels with various factors associated with CLD was also seen by independent sample t-test. Association between OPG levels and various signs and symptoms of CLD was checked by binary logistic regression analysis, where signs and symptoms were taken as dichotomous variable. Results of logistic regression were presented as OR. P<0.05 was considered statistically significant.

## RESULTS

The general, anthropometric measures and OPG levels of controls and cases are given in Table-I. The mean (SD) age of controls and cases in this study were 54.14 (8.145) and 53.30 (8.746) respectively. The mean (SD) levels of serum OPG in controls 3.99 (1.427) and cases 3.79 (1.563) were not significantly different (p = 0.615). The severity of disease was assessed by Child-Pugh classification. OPG levels were significantly different between the three Child-Pugh classes (p = 0.029). Tukey’s post hoc analysis showed that OPG levels were significantly high (p = 0.028) in class C, 4.356 (2.065) putting it under the umbrella of severe CLD as compared to class A, 2.832 (0.722) as shown in Table-II. In cases, OPG levels were not significantly different between having positive family history/ no positive family history group (p = 0.979) considering it as an independent variable of family history, ascites/ non-ascites group (p = 0.271), use of diuretics/ no use of diuretics group (p = 0.144), sodium restricted diet/no sodium restricted diet group (p = 0.958), smoker/ non-smoker group (p = 0.844), IV drug abuser/ non- abuser group (p = 0.841), diabetic/ non-diabetic group (p = 0.961), heart failure/ non-heart failure group (p = 0.726), renal disease/ non renal disease group (p = 0.077), cancer/ non-cancer group (p = 0.417) and cases doing daily walk/no walk group (p = 0.857) (Table-III). The binary logistic regression analysis showed that in CLD cases, there was a positive association of OPG with splenomegaly (OR = 2.10, p = 0.009), hepatomegaly (OR = 4.41, p = 0.012), skin pigmentation (OR = 2.06, p = 0.028), malena (OR = 1.87, p = 0.018), and prolong bleeding (OR = 1.86, p = 0.023). Table-IV.

**Table-I:** General, anthropometric characters and OPG levels in cases and controls.

	Control (Normal) n=29 Mean (SD)	Cases (CLD) n=50 Mean (SD)	P value
Age	54.14 (8.145)	53.30 (8.746)	0.675
Height (m)	1.56 (0.063)	1.58 (0.074)	0.082
Weight (kg)	66.93 (10.222)	75.62 (17.083)	0.015*
BMI	27.69 (4.900)	30.16 (6.768)	0.090
Waist circumference (cm)	78.28 (6.430)	84.22 (12.616)	0.021*
Hip circumference (cm)	86.03 (5.241)	94.88 (13.573)	0.001*
W/H ratio	0.92 ( 0.060)	0.89 (0.069)	0.033*
Ascites free weight (kg)	66.93 (10.222)	69.20 (24.571)	0.637
Pulse	85.72 (7.959)	80.92 (6.919)	0.006*
Systolic BP (mm Hg)	121.38 (14.072)	126.40 (14.813)	0.143
Diastolic BP (mm Hg)	80.34 (10.171)	82.80 (10.698)	0.320
Temperature	98.10 (0.310)	98.29 (0.701)	0.175
Respiratory rate	16.76 (2.415)	15.36 (2.405)	0.015*
OPG levels	3.99 (1.427)	3.79 (1.563)	0.615

* p < 0.05 is considered statistically significant.

**Table-II:** Severity of disease association with OPG using ANOVA.

Child-Pugh classification	OPG Levels		P value

	Mean	SD	
Class A (n=13)	2.832	0.722	0.029*
Class B(n=19)	4.017	1.139	
Class C(n=18)	4.356	2.065

Post hoc analysis Tukey HSD			

Groups	Mean difference	SEM	P value
Class A vs Class B	-1.184	0.567	0.105
Class A vs Class C	-1.523	0.567	0.028*
Class B vs Class C	-0.339	0.534	0.802

* p < 0.05 is considered statistically significant.

**Table-III:** Comparison of OPG levels with factors associated with CLD.

Variables		OPG levels		p-value

		Mean	SD	
Positive family history	Yes (n=16)	3.54	1.92	0.979
	No (n=34)	3.53	1.38	
Ascites	Yes (n=40)	3.65	1.66	0.271
	No (n=10)	3.04	0.98	
Use of Diuretics	Yes (n=24)	3.87	2.02	0.144
	No (n=26)	3.22	0.87	
Sodium restricted diet	Yes (n=44)	3.53	1.62	0.958
	No (n=6)	3.56	1.02	
Smoking	Yes (n=14)	3.46	1.24	0.844
	No (n=36	3.56	1.67	
IV drug abuse	Yes (n=2)	3.75	2.10	0.841
	No (n=48)	3.52	1.56	
Accidental skin prick	Yes (n=13)	4.01	1.57	0.206
	No (n=37)	3.37	1.54	
Diabetes Mellitus	Yes (n=24)	3.52	1.69	0.961
	No (n=26)	3.54	1.45	
Heart failure	Yes (n=3)	3.22	1.82	0.726
	No (n=47)	3.55	1.56	
Renal disease	Yes (n=4)	4.85	3.02	0.077
	No (n=46)	3.42	1.36	
Cancer	Yes (n=3)	2.82	0.33	0.417
	No (n=47)	3.58	1.59	
Daily walk	Yes (n=10)	3.61	2.21	0.857
	No (n=40)	3.51	1.38	

*p < 0.05 is considered statistically significant.

**Table-IV:** Association of OPG with signs and symptoms of CLD.

Signs and symptoms of CLD (no/ yes)	B^a^	Exp (B)^b^	p-value	95% CI
Splenomegaly	0.74	2.10	0.009*	1.20-3.69
Hepatomegaly	1.48	4.41	0.012*	1.37-14.13
Clubbing	-0.42	0.66	0.377	0.26-1.66
Pigmentation	0.72	2.06	0.028*	1.08-3.94
Menstrual disturbance/ Amenorrhea	0.57	1.78	0.054	0.99-3.19
Hemoptysis	-1.66	0.19	0.365	0.005-6.91
Hematemesis	0.34	1.40	0.121	0.91-2.17
Malena	0.63	1.87	0.018*	1.11-3.15
Epistaxis	0.31	1.36	0.218	0.83-2.22
Purpura	0.25	1.29	0.254	0.83-2.00
Petechiae	0.31	1.35	0.178	0.87-2.16
Bruisability	0.13	1.14	0.524	0.75-1.73
Prolong bleeding	0.62	1.86	0.023*	1.09-3.16

* p < 0.05 is considered statistically significant.

a **B** – This is the coefficient for the constant (also called the “intercept”) in the null mode.

b **Exp(B)** – This is the exponentiation of the B coefficient, which is an odds ratio.

## DISCUSSION

Cirrhosis is one of the last manifestation of chronic liver disease and is the leading cause of death in such patients. We investigated the serum levels of osteoprotegerin and its association with chronic liver disease especially in relevance to female patients. In the present study, we found a stepwise increase in serum OPG in patients with liver cirrhosis due to hepatitis B and hepatitis C, according to P-Ch scores (A, B, C). The increase was significant amongst class A versus C of Child-Pugh’s classification. We could not found significant difference between mean OPG levels in healthy control and cases. In line with our results, one study found an increase level of OPG in Child-Pugh classification C as compare to A. In their study, they took 30 cirrohotic patients and serological markers were positive for hepatitis B and C. OPG levels in Child–Pugh A patients was 5.1 pmol/l; range 4.8–5.2 which were significantly lower than those of patients in Group C 6.5 pmol/l; range 4.8–8.4).^14^ The reason for this increase in OPG levels in cirrhotic liver was linked to TGFβ.

In a recently conducted research on mice, they induced cirrhosis in the mice liver with CCl_4_. The precision cut liver slices of the mice confirmed that on stimulation with TGFβ liver tissue produces OPG. Furthermore, they also stimulated 3T3 fibroblast, which secretes TGFβ that was the main source of stimulation for OPG. Scientist documented that liver injury due to CCl_4_ causes activation of inflammatory cytokines such as TGFβ, which causes increase secretion of OPG. Further, strengthening their experiment, they remove the toxic stimuli of CCl4 and treatment with interferon alpha not only reversed the fibrosis but also decreased the expression of OPG levels.^15^ In contrast to our study, another study could not found any significant association between Child-Pugh classification and OPG levels. However, they do find significantly increase levels of OPG in cirrohotic patients as compare to control.^16^ We also compare levels of OPG with sign and symptoms of chronic liver disease, and found a significant positive association of OPG with splenomegaly, hepatomegaly, skin pigmentation, malena, and prolong bleeding. The reason could be as these sign, symptoms appear at later stages of the disease, and OPG levels were high as the severity of the disease rises according to Child-Pugh’s classification. Although scarce data is available linking OPG with hepatomegaly. In another study we found an increase levels of OPG in non-alcoholic fatty liver disease in which liver is usually enlarged.^17^ Similarly in another study OPG levels were positively associated with fat content of the liver and researchers were of the view that OPG might play a significant role in development of various liver diseases.^18^

In contrast another study shows a negative relationship of OPG with NAFLD.^19^ The reason for this difference could be that single serum biomarker can only show one aspect of disease progression and there are several modifiable and non-modifiable factors that are responsible for the overall situation. In our study there was not a significant difference of serum Osteoprotegerin levels between cases and controls. As this study includes only female participants and Estrogen can increase Osteoprotegerin levels in control females as supported by another study that estrogen may stimulate OPG expression in various cells at the transcriptional level. The role of microRNA (miRNA) in estrogenmediated OPG production in human osteoblastlike MG63 cells was investigated in this study. The results from ELISA, western blotting and reverse transcription-quantitative polymerase chain reaction (RTqPCR) confirmed that estrogen may upregulate OPG expression.^20^ Estrogen inhibits bone resorption by inducing changes in multiple estrogen-dependent regulatory factors including TNF-α and the OPG/RANKL/RANK system.^21^ In another study Serum levels of osteoprotegerin were seen to have a significant positive association with bone mineral density at the lumbar spine, at the femoral neck, total hip in women using estrogen as compared to non-users of estrogen.^22^

### Limitation of the study

It includes single center study and small sample size was another limitation of our study.

## CONCLUSION

The levels of OPG, an inflammatory cytokine is increased in chronic liver disease Child-Pughs C classification and it can be of potential use as a biomarker to diagnose the severity of the disease in such patients.

### Authors’ Contribution:

**Saba Tariq:** Designed the study, data entry, manuscript writing. She is also responsible for the accuracy or integrity of the work.

**Sundus Tariq:** Statistical analysis and interpretation of data.

**Shaista Hussain:** Data Collection and drafted the manuscript.

**Mukhtiar Baig:** Review and final approval of the manuscript.
